# Beyond Seizures: A Comprehensive Review of Giant Somatosensory Evoked Potentials

**DOI:** 10.3390/jcm14165755

**Published:** 2025-08-14

**Authors:** Giuseppe Magro

**Affiliations:** Department of Neurology, Lamezia Terme Hospital, 88100 Catanzaro, Italy; giuseppemagro.neuro@gmail.com

**Keywords:** somatosensory evoked potentials, somatosensory cortex, cortical hyperexcitability, giant SEP, giant somatosensory evoked potentials, increase in amplitude somatosensory evoked potentials

## Abstract

Giant somatosensory evoked potentials (gSEPs) are abnormally high-amplitude cortical responses to peripheral nerve stimulation, traditionally regarded as electrophysiological hallmarks of progressive myoclonic epilepsies (PMEs). However, accumulating evidence shows their presence in a broader range of non-epileptic conditions, including focal lesions, metabolic encephalopathies, neurodegenerative diseases, and even functional disorders. This review offers a comprehensive analysis of the physiological mechanisms, diagnostic criteria, and clinical significance of gSEPs, integrating data from both classical and emerging neurophysiological techniques. gSEPs are mainly produced in the primary somatosensory cortex through mechanisms involving cortical disinhibition, impaired GABAergic transmission, and altered thalamocortical connectivity. In epileptic syndromes such as Unverricht–Lundborg disease and other PMEs, gSEPs reflect cortical hyperexcitability and are closely linked to cortical myoclonus. Conversely, in non-epileptic contexts, they may indicate transient or chronic cortical dysfunction. The diagnostic utility of gSEPs ranges from differential diagnosis of myoclonus to monitoring disease. However, heterogeneity in amplitude definitions and recording protocols hinders the standardization of these measurements. This may result in the identification of the right threshold to differentiate conditions associated with simple increased versus giant SEP, the latter of which may help identify truly epileptic conditions from other disorders simply associated with increased SEP amplitude.

## 1. Introduction

Somatosensory evoked potentials (SEPs) are electrophysiological responses elicited by peripheral nerve stimulation and recorded from the scalp in a time- and phase-locked manner. They are a valuable tool for evaluating the functional integrity of the dorsal column–medial lemniscal sensory pathways. The median nerve is the most frequently stimulated site in clinical and research settings. Cortical responses are typically recorded over the parietal scalp. They are characterized by a sequence of waves: an initial negative deflection around 20 milliseconds (N20) and a subsequent positive wave at approximately 25 milliseconds (P25), both generated in the primary somatosensory cortex (postcentral gyrus). These are often followed by a later component around 30–35 milliseconds (N30–N33/35), which may reflect contributions from the precentral motor cortex or associated areas [1]. They function as diagnostic biomarkers and tools for exploring the integrity and plasticity of cortical networks. Their increased clinical use in the last twenty years has provided new insights into their generation, disease distribution, and prognostic significance. In clinical neurophysiology, SEPs are employed to evaluate the integrity of the somatosensory pathways [2]. Increased amplitude SEPs are often referred to as giant somatosensory evoked potentials (gSEPs), initially described by Dawson [3], and have emerged as valuable and potential indicators of cortical hyperexcitability. In gSEPs, the P25 and N35 components are abnormally enlarged, while N20 is usually not. These responses were initially observed in patients with progressive myoclonic epilepsies (PMEs) and have since become crucial to the neurophysiological evaluation of individuals with myoclonus and other epileptic syndromes [4]. In truth, gSEPs have become a biomarker for cortical hyperexcitability across various disorders. The history of gSEPs dates back to the early 1980s when they were first reported in patients with Unverricht–Lundborg disease (ULD). Subsequent work by Shibasaki and colleagues defined the electrophysiological signature of gSEPs [4,5]. Their consistent presence in PMEs established them as a diagnostic hallmark. In clinical practice, detecting gSEPs during somatosensory evoked potential studies often requires a more thorough evaluation of genetic epilepsies or neurodegenerative conditions. Beyond their diagnostic value, studying gSEPs has significantly enhanced our understanding of cortical excitability and inhibitory failure in the human brain. Their identification has become central to diagnosing Unverricht–Lundborg disease and Lafora disease. Canafoglia et al. confirmed the clinical relevance of gSEPs in myoclonic syndromes, where these signals serve as both a diagnostic and prognostic marker [6]. Their abnormally high amplitude distinguishes giant SEPs, but there is no clear consensus on the definition of gSEP [7]. Shibasaki et al. originally defined SEPs as giant when the amplitude of the P25 exceeded 8.6 μV or the N33 surpassed 8.4 μV, based on comparative data from healthy individuals and patients with various cerebral lesions [5]. A more commonly adopted criterion in the literature considers an amplitude greater than the mean plus two to three standard deviations of control subjects [6,8]. In contrast, other studies employ arbitrary thresholds, such as 10 μV [8] or do not specify a cut-off. Additionally, some authors require an increased amplitude of the N20–P25 and P25–N33 components to define a giant SEP, whereas others accept enlargement of only one of these components as sufficient [7]. Moreover, there is no consistency in measuring SEP amplitude (peak-to-peak or baseline-to-peak). There is also high variability within the same patient, for example, during and off-therapy. Many have suggested the possible influence of anti-seizure medications, such as Perampanel [9]. Other authors, such as Rothwell and colleagues, suggested there was no effect on SEPs’ amplitude after clonazepam [10]. Table 1 summarizes the currently available definition of gSEP.

Despite their role in recognizing PMEs, gSEPs have been described in various non-epileptic syndromes, challenging the initial view limited to cortical hyperexcitability [23,24]. Moreover, the recording condition of gSEPs is different from that of short-latency SEPs [12,13,14,25]. In a controlled study with healthy subjects, Demura et al. compared the waveform features of SEPs recorded under standard short-latency SEP (SSEP) conditions with those obtained using gSEP protocols. Simultaneous recordings from 24 adults showed that the N20–P25 component’s amplitude was significantly higher in gSEP than in SSEP (10.0 µV vs. 7.8 µV), averaging about an 80% increase. A strong linear correlation was found between the two modalities, indicating that an N20–P25 amplitude of ≥8 µV in SSEP could relate to the diagnostic threshold of ≥10 µV in gSEP. These results suggest that SSEP, which is more feasible in clinical settings, could be a useful initial screening tool for cortical hyperexcitability without needing the low-frequency stimulation or broad bandpass filters that are typical for gSEP. This study supports using SSEP and gSEP in a complementary, graded manner to detect and monitor cortical hyperexcitability across various neurological conditions, including progressive myoclonic epilepsies.

## 2. Neurophysiological Basis of gSEPs

### 2.1. Preclinical Animal Model of Giant Somatosensory Evoked Potentials

Preclinical animal models have significantly advanced our understanding of SEPs and gSEPs, providing insights that translate into clinical applications related to cortical hyperexcitability. Pigs, specifically, have become valuable models because of their neuroanatomical similarities to humans. In a feasibility study, Hoareau et al. demonstrated that high-quality SEPs could be reliably recorded in pigs using a non-invasive EEG and light sedation, avoiding the confounding effects of general anesthesia [26]. Building on this, Hilgart et al. recorded P30 components of SEPs in a porcine model of chronic neuropathic pain. They found significant latency prolongation in injured limbs, supporting the use of SEP latency as a cortical biomarker of sensory processing deficits [27]. Similarly, Cui et al. carried out a cross-species analysis involving rats, goats, and humans, revealing strong linear correlations between SEP time–frequency components across these species. Importantly, stable distributions of main and sub-components were maintained even after spinal cord injury, highlighting the robustness of SEP architecture and its potential to indicate pathological changes in central pathways [28].

Rodent studies have also uncovered abnormal SEP dynamics in conditions involving cortical plasticity and dopaminergic dysregulation. For instance, dopamine transporter knockout (DAT-KO) rats, a well-established model of hyperdopaminergia, exhibit desynchronized activity between the striatum and prefrontal cortex during active behaviors, with decreased power in striatal local field potentials [29]. These electrophysiological disruptions mirror clinical findings in disorders associated with dopamine imbalance, such as Parkinson’s disease. Additional evidence from animal epilepsy models further supports the importance of these circuits and temperature in SEP amplitude variations [29,30]. These studies collectively highlight the importance of animal models—not only in verifying the physiological basis of gSEPs but also in identifying electrophysiological markers of cortical excitability that reflect human pathophysiology. Future research could clarify whether giant-like SEPs in animals truly indicate hyperexcitable cortical states and how these states relate to epileptic and non-epileptic conditions observed in clinical neurology.

### 2.2. Human Studies and Pathophysiology of Giant Somatosensory Evoked Potentials

The emergence of gSEPs is believed to be fundamentally linked to modifications in cortical excitability, particularly concerning the primary somatosensory cortex. Neurophysiological investigations suggest that augmented excitatory neurotransmission, combined with insufficient inhibition, underpins the observed phenomena. The dysfunction of GABAergic interneurons, elevated glutamatergic drive, and altered thalamocortical inputs collectively generate gSEPs. Depending on the underlying disease, these mechanisms may arise in the context of genetic mutations, metabolic defects, or degenerative processes [4]. The source of gSEPs has been suggested to lie in the primary somatosensory area (S1), especially in the 3b area, given the latency similar to that of normal SEPs. Other putative involved areas include area 3a, which receives proprioceptive afferents, as well as the primary motor cortex and supplementary motor areas [15,31]. Some authors have noted that the initial N20 component of the gSEP is not enlarged, possibly indicating that the initial thalamocortical input is processed normally. Instead, the later components (P25–N33) are enlarged, thought to reflect the activity of area 1, which receives input from area 3b [10]. Many hypotheses have been made to explain the pathogenesis of gSEP. The lack of inhibition seems to be the most important underlying mechanism [15]. This may result from reduced cerebellar inhibition [32] or abnormal firing neurons [33]. A thalamic contribution to the gSEP, besides cortical hyperexcitability, has been hypothesized, and it is supported by evidence of abnormal SEP-related high-frequency oscillations (HFOs) in some myoclonic epilepsies [34,35]. This observation has led some authors to hypothesize that gSEP might be related to the dysfunction of GABAergic interneurons in the cortical sensory motor network. Transcranial magnetic stimulation studies also point to reduced sensorimotor cortical inhibition [36,37]. The pathophysiology of giant SEP is still under investigation. Nonetheless, the presumed localization of gSEP in the S1 area can explain why there is not always a corresponding myoclonic jerk following the abnormal sensory cortex activity [7]. Emerging electrophysiological technologies, including magnetoencephalography (MEG) and high-density EEG, could help provide more accurate spatial localization of the cortical generators of gSEPs [38,39]. MEG enables more precise localization of cortical sources of somatosensory evoked fields, especially the N20 and P25 components from the sensorimotor cortex. Although it is not specific to giant SEPs, MEG research has shown its capability to resolve the spatial and temporal patterns of early cortical responses to somatosensory input, indicating its potential to assess cortical hyperexcitability in myoclonic disorders [40]. Recent advances in ultra-high-density EEG (uHD EEG) technology have achieved unprecedented spatial resolution in mapping SSEP. A recent study showed that uHD EEG can identify the central sulcus with over 95% accuracy using SSEP topography, nearly matching the precision of invasive intracranial recordings [41]. Although not intended to examine pathological SEPs, this approach underscores the potential of high-resolution EEG in detecting subtle cortical dynamics. Although the primary interpretation of gSEPs is that they reflect exaggerated sensorimotor cortical excitability, emerging evidence suggests that broader networks—including thalamocortical circuits and prefrontal regions—may contribute to sensory processing and SEP modulation. Studies in both healthy subjects and patients with epilepsy have highlighted the influence of cognitive and attentional factors on SEP amplitude. Cortical disinhibition, particularly through impaired GABAergic transmission, is a key mechanism supported by neurophysiological models [42].

Beyond cortical disinhibition and impaired GABAergic transmission, recent studies suggest that gSEPs may reflect surface signs of paroxysmal depolarization shifts (PDSs) within hyperexcitable cortical networks. Shibasaki et al. first showed that cortical spikes before myoclonic jerks follow a spatiotemporal pattern with gSEPs, indicating a common origin [4,5]. This idea has been further clarified by time–frequency analysis electrophysiology studies, which distinguish short-latency components (SLCs) as excitatory PDS-like events and mid-latency components (MLCs) as their inhibitory afterpotentials [42].

Recent multimodal imaging studies offer additional insights. In patients with PMEs, simultaneous EEG-fMRI has shown dissociated neurovascular responses. While SEP amplitudes are abnormally high, BOLD activity in the sensorimotor cortex is paradoxically decreased, suggesting a loss of thalamocortical gating and compensatory subcortical recruitment [21]. This reinforces the idea that gSEPs reflect abnormal network-level dynamics beyond focal cortical excitation. At the synaptic level, presynaptic mechanisms also seem essential. GABAergic presynaptic inhibition—through axo-axonic synapses—modulates incoming input by shunting glutamatergic transmission. This process has been extensively studied in spinal systems and is now thought to play a role in cortical sensory pathways [43]. Recent evidence suggests that in cortical myoclonus, impaired cerebello-thalamo-cortical inhibition may paradoxically enhance cortical excitability. Anodal cerebellar stimulation in patients increased SEP amplitudes and reduced intracortical inhibition, especially in those with giant SEPs, highlighting a dysfunctional cerebellar modulation of sensorimotor circuits [44]. Disruption of these mechanisms may cause excessive afferent gain, increasing the somatosensory cortical responses.

Furthermore, an imbalance in the excitation/inhibition ratio is increasingly seen as a common mechanism in hyperexcitable states. A recent review highlighted that dynamic dysregulation of the GABA/glutamate ratio contributes to cortical instability, especially in epileptic syndromes [45,46]. Therefore, gSEPs may serve not only as a marker of cortical disinhibition but also as a downstream indicator of multiple interconnected disruptions across synaptic networks.

## 3. Giant Somatosensory Evoked Potentials in Epileptic Conditions

**Figure 1:**
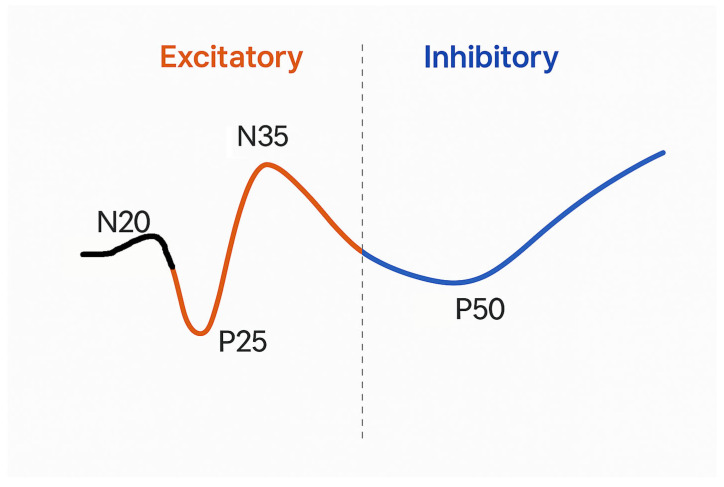
Schematic diagram of a giant somatosensory evoked potential (gSEP) waveform illustrating excitatory and inhibitory parts. The waveform, triggered by median nerve stimulation, features a small initial N20 deflection followed by the main excitatory components: P25 (positive peak) and N35 (negative peak). These are interpreted as stimulus-induced paroxysmal depolarization shifts (PDS-like activity), indicating cortical hyperexcitability. The following P50 is marked as an inhibitory component, identified by a decrease in high-frequency power and viewed as post-excitatory suppression or cortical hyperpolarization. Excitatory components are highlighted in orange, while the inhibitory segment appears in blue. A dashed vertical line separates the excitatory phase from the inhibitory phase.

gSEPs are a hallmark of different epileptic syndromes characterized by cortical myoclonus and sensorimotor hyperexcitability. These waveforms, typically evoked by median nerve stimulation, display abnormally increased amplitude, most prominently in the P25 and N35 components, and are consistently recorded in patients with PMEs such as ULD, benign adult familial myoclonus epilepsy (BAFME), Lafora disease, sialidosis, and myoclonic epilepsy with ragged-red fibers (MERRF) [4,33,42]. The presence of gSEPs is not merely an epiphenomenon of myoclonus but represents a core electrophysiological feature of cortical hyperexcitability in epilepsy. In classical studies, Shibasaki and colleagues demonstrated that the P25 component of gSEP is time-locked with the cortical spike preceding myoclonic jerks in jerk-locked back averaging (JLA), suggesting its equivalence to an evoked PDS—a key element in epileptogenesis [4]. More recent data using high-resolution time–frequency analysis have further refined this model: P25 and N35 are now considered to reflect the excitatory epileptic complex, while the P50 component is interpreted as a subsequent inhibitory wave, mimicking the suppression phase seen after interictal spikes [42]. In patients with BAFME, gSEPs are often accompanied by HFOs superimposed on the P25 peak (P25-HFOs), a finding of diagnostic and pathophysiological significance. HFOs are known biomarkers of epileptic tissue and are particularly prominent in BAFME, where they correlate with the characteristic autosomal-dominant cortical myoclonus and may reflect disinhibited activity within the primary sensorimotor cortex [47]. Notably, in a study on BAFME, authors showed that gSEPs were highly reproducible across subjects and could be reliably elicited through standard median nerve stimulation, with P25 amplitudes significantly surpassing normative thresholds. Additionally, the amplitude of gSEPs exhibited a strong correlation with the presence of cortical reflex myoclonus. Interestingly, while most affected individuals displayed both gSEPs and C-reflexes, a minority—especially younger patients or those in early disease stages—lacked one or both findings, indicating potential timing variability or incomplete phenotypic expression. These findings reinforce the clinical value of gSEPs in evaluating suspected BAFME, particularly in distinguishing it from functional or subcortical mimics, and suggest their role as trait markers of sensorimotor cortex disinhibition in familial cortical myoclonus [48]. The sensitivity of gSEPs to pharmacological intervention further supports their epileptic origin. Treatment with perampanel, an AMPA receptor antagonist, significantly reduces the amplitude and extends the latency of the SLC components (P25/N35), without impacting the inhibitory middle latency component (P50). This supports the idea that P25/N35 are related to glutamate-driven paroxysmal depolarization shift-like events, while P50 indicates GABAergic suppression [9,42]. Beyond PMEs, gSEPs have been variably reported in patients with generalized epilepsy, focal epilepsies, and epilepsia partialis continua. However, their diagnostic utility in these settings is less robust and often confounded by technical or comorbid variables [49]. Figure 1 summarizes the excitatory and inhibitory components of gSEPs.

In acute encephalopathic states, including post-anoxic and post-surgical coma, transiently enlarged gSEPs have been observed alongside epileptiform discharges on EEG; their subsequent normalization has coincided with clinical improvement, while persistence has been linked to poor prognosis, indicating their potential—when interpreted with EEG—as dynamic markers of cortical excitability and recovery potential [50].

Table 2 summarizes the key features of gSEP in the most commonly encountered PMEs.

In summary, gSEPs in epileptic conditions are not simply exaggerated sensory responses but reflect complex epileptic evoked potentials with distinct excitatory and inhibitory phases. Their interpretation benefits from integrative approaches combining waveform morphology, time–frequency analysis, pharmacological sensitivity, and clinical correlation. Future research may explore whether these potentials can serve as quantitative biomarkers for treatment monitoring or for distinguishing between epileptic and functional myoclonus.

## 4. Giant Somatosensory Evoked Potentials in Non-Epileptic Disorders

Although gSEPs are most commonly associated with epileptic syndromes, particularly those involving cortical myoclonus, they have also been reported in several non-epileptic neurological disorders, often raising essential questions about their underlying mechanisms and clinical significance. In these contexts, gSEPs appear to reflect cortical hyperexcitability that is not necessarily epileptogenic but may result from disinhibitory, degenerative, or maladaptive plastic processes. Table 3 summarizes commonly encountered non-epileptic disorders associated with gSEP.

### 4.1. Neurodegenerative Diseases

In a study of eight patients with Creutzfeldt–Jakob disease (CJD), SSEPs were examined at different disease stages. While early and mid-stage patients showed normal SSEPs despite abnormal EEGs, later stages demonstrated a progressive decrease and eventual disappearance of the N20 component, without changes in latency. Notably, giant SSEPs were observed in two of three patients in the later stage, consistent with a decline in EEG periodic sharp wave complexes. These gSEPs lessened as the disease progressed, suggesting a phase-dependent cortical hyperexcitability followed by neuronal loss. The results show that while SSEP amplitude, especially N20, stays stable in early stages, it gradually declines with cortical degeneration, reflecting the pathological progression of CJD [54]. The presence of enlarged somatosensory evoked potentials significantly correlated with the myoclonic presentation in some studies [55,56]. Isolated cases of negative myoclonus also showed gSEPs, linked to increased C-reflexes and periodic EEG discharges, indicating a temporary boost in cortical excitability followed by suppression. Therefore, while gSEPs may appear temporarily in some mid-stage CJD patients, they do not consistently match characteristic EEG patterns or myoclonic movements and likely represent a brief period of cortical disinhibition before widespread cortical failure [54,56,57].

Numerous reports in the literature document large-amplitude evoked potentials in MSA and PSP. This indicates that evoked potentials might serve as an early distinguishing feature between PSP and Parkinson’s disease [58]. Several reports have shown a high prevalence of gSEP in PSP and MSA; however, the mechanism remains uncertain. Many have suggested subcortical disinhibition as the underlying cause [24,59].

### 4.2. Focal Structural Lesions

gSEPs have been observed in patients with glioblastoma and other focal cortical lesions, especially when the rolandic cortex or paracentral lobule is affected. In these cases, gSEPs are often unilateral, indicating localized cortical disinhibition near the lesion. For example, Alsallom et al. reported a case of a patient with a right frontal glioblastoma where left median nerve stimulation caused giant cortical components (N20/P22 amplitude of 15.3 μV, about four times larger than in the opposite hemisphere). Interestingly, the gSEPs disappeared after surgical removal of the tumor, suggesting they could serve as functional biomarkers of cortical instability before motor symptoms or seizures occur [60]. Vascular etiology was the most common condition associated with large amplitude SEP in a large cohort of non-epileptic patients [24]. Interestingly, in some cases, the gSEP was reported to be contralateral to the central nervous system lesion, possibly underlying a maladaptive or compensatory mechanism [24,61]. Other structural causes include demyelination disorders such as multiple sclerosis, space-occupying lesions both in the brain and spinal cord, and cervical myelopathy [23,24].

### 4.3. Metabolic and Similar Conditions

gSEPs may rarely emerge transiently in metabolic conditions characterized by cortical hyperexcitability, even in the absence of primary epileptic disorders. A paradigmatic example is provided by a case of hypocalcemia-induced cortical myoclonus reported in an elderly woman with a history of total thyroidectomy 12 years prior. The patient presented with multifocal myoclonic jerks predominantly affecting the upper limbs, jaw, and face. Neurophysiological investigations revealed gSEPs, pathologically enhanced long-latency reflexes, and a cortical potential preceding the jerks on jerk-locked back averaging, establishing a cortical origin of the myoclonus. Notably, all clinical and electrophysiological abnormalities resolved completely following correction of serum calcium levels, underscoring the reversible nature of the cortical dysfunction. This case illustrates that gSEPs can serve as a biomarker of transient cortical hyperexcitability secondary to metabolic derangements such as post-surgical hypocalcemia. It highlights the importance of considering non-epileptic etiologies in the differential diagnosis of gSEP-related symptoms and signs [62]. Other metabolic conditions associated with gSEP include vitamin B12 deficiency and hydroxychloroquine poisoning [24].

### 4.4. Functional and Other Non-Epileptic Disorders

gSEPs, traditionally associated with cortical myoclonus and epileptic cortico-sensory syndromes, may also be observed in non-myoclonic and functional neurological conditions, including functional neurological disorders (FND). Rossi Sebastiano et al. reported that gSEPs are not specific to myoclonic disorders, as they were identified in a variety of non-epileptic conditions such as multiple sclerosis, progressive supranuclear palsy, motor neuron disease, chronic pain, and even functional disorders [23]. These findings likely reflect a spectrum of underlying pathophysiological mechanisms, from cortical disinhibition due to deafferentation (as in multiple sclerosis, stroke, or spinal cord diseases), to early cortical hyperexcitability in degenerative conditions such as motor neuron disease. Although it remains unclear whether these gSEPs in FND consistently reach the formal diagnostic amplitude threshold, their presence suggests a transient sensitization or maladaptive plasticity of the sensorimotor network, independent of cortical myoclonus or overt epilepsy. Altered central somatosensory processing in chronic pain functional patients has been documented without SEP abnormalities [63]. Moreover, recent evidence suggests that pain in FND possibly reflects a descending pain inhibitory control impairment, providing arguments for a possible lack of central inhibition in the generation of excessive response to sensory stimulus [64]. Among different forms of FND, functional tremor, functional myoclonus, and functional dystonia, gSEPs have been associated with functional myoclonus the most, where they may reflect heightened cortical maladaptive responses [65].

The presence of gSEPs in non-epileptic disorders highlights their value as non-invasive markers of cortical hyperexcitability, even when clinical seizures are absent. Their detection should lead clinicians to consider a broader differential diagnosis that includes neurodegenerative, metabolic, autoimmune, and structural causes. 

## 5. Clinical Utility and Diagnostic Applications

The presence of gSEPs indicates increased cortical excitability and can help differentiate cortical from subcortical myoclonus, alongside C-reflex and JLA. In patients with unclear, jerky movements, gSEPs provide confirmatory evidence of cortical origin, particularly when supported by EEG and EMG back averaging. This is crucial for differentiating PME and related disorders from non-epileptic or subcortical mimics.

### 5.1. Applications in Critical Care and Coma Prognostication

In intensive care settings, gSEPs may aid in evaluating comatose patients with post-anoxic encephalopathy [66] or non-convulsive status epilepticus [67]. Absent and very low amplitude SSEPs appear to be highly predictive of poor outcome after cardiac arrest [68,69]. However, their usefulness is limited by factors such as sedation, hypothermia, or underlying metabolic imbalances [50]. Conversely, the presence of giant or paradoxically large SEPs in comatose patients may suggest preserved or disinhibited cortical reactivity, although this area requires further exploration.

### 5.2. Early Detection and Longitudinal Monitoring

In the outpatient setting, gSEPs aid in early detection of PME, guiding genetic testing and family counseling [4]. Moreover, tracking changes in SEP amplitude can assist in monitoring disease progression or response to anti-seizure treatment, although comprehensive longitudinal data are still limited. In a longitudinal study, SSEP amplitudes of P25 and N35 were enlarged in all patients and were gradually decreased with aging in ULD on average. The degree of temporal changes of P25 and N35 in ULD was similar to or even lower than that of healthy subjects [70]. Moreover, electrophysiologic tests such as SSEP may help detect other affected siblings before symptoms appear, once an index case is identified [71].

### 5.3. Neurorehabilitation and Cortical Plasticity

In rehabilitation, gSEPs might serve as neurophysiological markers of cortical plasticity, especially for patients undergoing therapy for post-stroke motor recovery or traumatic brain injury. Changes in SEP amplitudes during neurorehabilitation interventions may help monitor clinical progress, although few studies are available in this area [72]. For example, it is intriguing to speculate that the interhemispheric difference in SEP’s amplitude may serve as a monitor tool of response to rehabilitation: the maladaptive contralateral SEP amplitude minus the amplitude on the affected side.

Standardizing SEP acquisition protocols and normative values is essential for maximizing their diagnostic effectiveness; however, a clear definition is still lacking. Using gSEPs as early diagnostic tools in young patients with unexplained tremors or jerks has gained interest and may be helpful, especially in imaging negative patients. Combining advanced imaging techniques, such as functional MRI or PET, may improve diagnostic accuracy when gSEPs alone are inconclusive. Emerging portable devices and ambulatory SEP recordings could transform access to gSEP assessments in outpatient or resource-limited settings. A practical ambulatory screening tool to detect gSEPs could serve as a shortcut to therapy and the proper diagnostic pathway. These technologies may have the potential for screening at-risk populations, enabling real-time monitoring of cortical excitability, and supporting longitudinal studies.

## 6. Diagnosis and Interpretation

gSEPs must always be interpreted in a multimodal clinical context to avoid misdiagnosis or overinterpretation. Differential diagnosis of gSEPs involves distinguishing true cortical hyperexcitability from technical artifacts or physiological variants. The clinician must consider the patient’s clinical presentation, EEG findings, and other neurophysiological tests to reach their diagnosis. Medications also appear to interfere with and modulate SEP amplitudes and must be accounted for, as previously discussed. In metabolic disorders such as hepatic or uremic encephalopathy, transient alterations of the SEP, including an increase in latency, may be observed, often resolving with correction of the underlying condition [73]. Interpreting gSEPs outside a multimodal clinical setting risks both overdiagnosis and misdiagnosis, especially in ambiguous cases. For example, Rossi Sebastiano et al. reported that increased-amplitude SEPs appeared in a diverse group of neurological patients, including those with multiple sclerosis, motor neuron disease, and even functional neurological disorders—conditions not typically linked to cortical myoclonus [23]. Without thorough clinical and electrophysiological correlation (such as the presence of C-reflexes, jerk-locked back-averaging findings, or EEG epileptiform discharges), simply detecting gSEPs could falsely indicate cortical hyperexcitability. Similarly, in critically ill patients, Houlden et al. observed gSEPs coinciding with epileptiform EEG activity in comatose individuals, warning that these findings might represent a temporary cortical irritability rather than a definitive sign of epilepsy or a poor outcome [50]. Additionally, functional disorders can sometimes show increased SEP amplitudes due to heightened attention or non-specific cortical sensitization, which further complicates interpretation when assessed alone [23]. These examples highlight that while gSEPs can be helpful, they should be integrated with clinical history, imaging, EMG-EEG correlation, and disease context to ensure precise diagnosis.

The differential interpretation of gSEPs must also consider somatosensory stimulus parameters such as frequency, intensity, temperature, and limb of stimulation. Upper limb stimulation typically yields larger cortical responses than lower limb stimulation due to somatotopic representation and signal dispersion. It could be said that SEP asymmetry may hint at focal cortical lesions, and concurrent neuroimaging should be reviewed in such contexts. Significant asymmetry could be more informative than gSEP, given the lack of consensus on the definition of gSEP in terms of amplitude.

**Figure 2:**
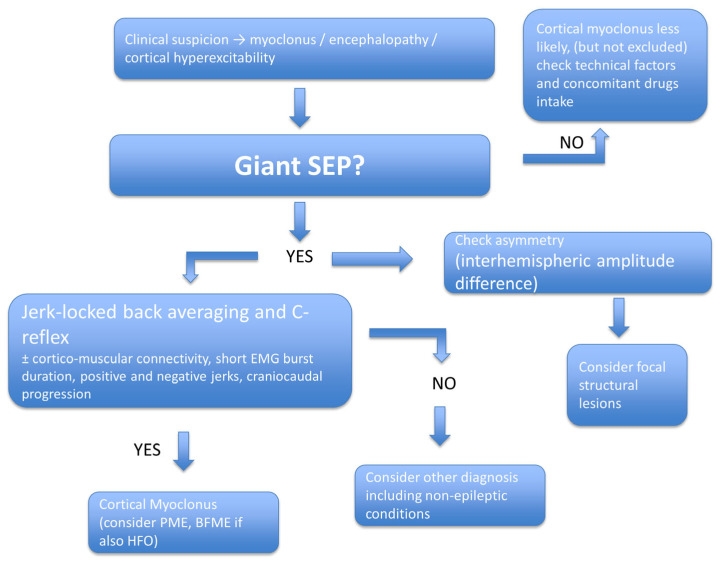
Stepwise clinical interpretation of giant somatosensory evoked potentials (gSEPs). This diagnostic flowchart provides a structured approach to interpreting gSEPs in patients presenting with myoclonus, encephalopathy, or suspected cortical hyperexcitability. The presence of gSEPs prompts further evaluation of interhemispheric asymmetry, jerk-locked back averaging (JLBA), C-reflex, cortico-muscular coherence (CMC), short EMG burst duration, and the presence of positive or negative myoclonic jerks. A consistent combination of these features supports the diagnosis of cortical myoclonus. It may help differentiate it from conditions like progressive myoclonic epilepsy (PME) or benign familial myoclonic epilepsy (BFME), mainly if high-frequency oscillations (HFOs) are also observed. Absence of gSEPs does not exclude cortical myoclonus and warrants careful assessment of technical quality, medications’ influence, and alternative diagnoses, including non-epileptic conditions or focal structural lesions.

The following flow diagram helps clinicians navigate the diagnostic challenges of cortical myoclonus (Figure 2).

## 7. Future Directions and Conclusions

The evolving landscape of neurophysiology and signal analysis presents new opportunities to utilize gSEPs as dynamic markers of cortical excitability. gSEPs have emerged as valuable, non-invasive markers of cortical hyperexcitability. Their detection can aid in differentiating cortical from subcortical myoclonus, support the diagnosis of PMEs, and reveal hidden cortical dysfunctions even in non-epileptic conditions. However, their interpretation requires careful integration with clinical, neurophysiological, and imaging data. Current limitations include the lack of standardized cutoffs, inter-laboratory variability, and an incomplete understanding of their prognostic value. Future studies should aim to establish precise amplitude cutoffs, correlate gSEPs with imaging biomarkers, and clarify their predictive value. In conclusion, gSEPs are a valuable tool in the diagnostic toolkit of clinical neurophysiology. However, their interpretation must be context-specific, multidisciplinary, and continually updated in light of new evidence. Moving forward, clinical trials may include gSEPs as outcome measures for therapies aimed at reducing cortical excitability. Genotype–phenotype relationships in PME could also be enhanced through SEP profiling. Furthermore, databases of normative SEP values categorized by age, sex, and stimulation protocol could help standardize practice worldwide. Although gSEPs are just one part of a broader neurophysiological assessment, their diagnostic and research potential continues to expand. Research into the genetic factors influencing gSEPs, such as polymorphisms in GABA-A receptor genes or glutamate transporter genes, might reveal predispositions to cortical hyperexcitability. Additionally, integrating EEG, SEP, MEG, and fMRI data could offer a comprehensive picture of cortical reactivity in health and disease. AI tools trained on large neurophysiological datasets may eventually identify subtle SEP signatures indicative of neurodegeneration, epilepsy onset, or treatment response. Looking ahead, integrating AI could significantly improve the diagnostic and prognostic capabilities of gSEPs. Machine learning algorithms may assist in distinguishing true gSEPs from physiologically elevated SEPs and decrease inter-operator variability through automated artifact removal and waveform classification. AI may help categorize evoked potentials by electrophysiological features, including SEP amplitude profiles, interhemispheric asymmetries, and high-frequency oscillations, which may support early detection of epileptic conditions or inform genetic testing. Additionally, AI-driven analysis of longitudinal SEP changes could facilitate early identification of disease progression or treatment response. Portable SEP systems with integrated AI could further expand these advantages to outpatient settings or resource-constrained environments.

Ultimately, the concept of ‘evoked potential phenotyping’ could enable clinicians to classify patients into distinct electrophysiological endotypes, supporting personalized treatment approaches. For example, in BAFME, the presence of P25-linked HFOs sets it apart from typical PMEs. Likewise, in post-anoxic coma, surprisingly giant SEPs may indicate the brain’s potential for cortical recovery. These phenotypes could serve as future biomarkers for prognosis and treatment response. Moreover, appropriate cut-offs may help distinguish between patients with SEP simply increased in amplitude versus patients with gSEP, the latter of which may be more suggestive of an epileptic condition. These considerations stem from the fact that SEPs, which increase in amplitude, cannot be considered an exclusive hallmark of myoclonus or epilepsy. Indeed, as suggested in previous works, it may represent a sign of adaptive, plastic, or degenerative cortical change. Longitudinal cohort studies are necessary to determine whether gSEP patterns change throughout disease progression and to understand their relationship with comorbidities.

## Figures and Tables

**Table 1:** Definition of giant somatosensory evoked potentials (gSEPs).

Authors and References	Definition of Giant SEPs (gSEPs)
Shibasaki et al., 1977 [5]	Giant SEP defined as P25 amplitude > 8.6 μV or N33 amplitude > 8.4 μV
Canafoglia et al., 2017; Caviness et al., 2002; Hitomi et al., 2011; Ikeda et al., 1995; So et al., 1989; Terada et al., 1997; Visani et al., 2013 [6,8,11,12,13,14,15]	SEP amplitude greater than the mean plus 2–3 standard deviations of healthy control subjects.
Rodriguez et al., 1994; Thompson et al., 1994c [16,17]	Threshold N20-P25 ≥ 10 μV.
Brown et al., 1991; Lu et al., 1998; Okuma et al., 2005; Storti et al., 2017; van Egmond et al., 2014 [18,19,20,21,22]	No clearly defined amplitude cut-off provided.
General variations	Some authors require increased amplitudes of both N20–P25 and P25–N33 components, while others consider enlargement of only one component sufficient.

**Table 2:** Key features of the most commonly encountered progressive myoclonic epilepsies (PMEs) and BAFME (benign adult familial myoclonus epilepsy). Legend. Unverricht–Lundborg disease: ULD; BAFME: benign adult familial myoclonus epilepsy; FAME: Familial Adult Myoclonic Epilepsy; MERRF: myoclonic epilepsy with ragged-red fibers; gSEP: giant somatosensory evoked potentials; HFOs: high-frequency oscillations; time-locked: myoclonus temporally correlated with SEP components or EEG spikes; NA: not available; μV: microvolts.

Syndrome	gSEP Frequency (%)	SEP Components (gSEP)	Typical Amplitude (μV)	SEP–Myoclonus Correlation	References
Unverricht–Lundborg (ULD)	~52%	N20–P25; P25-N33	N20–P25 22.0 ± 8.5 μV;	Mostly no	Visani [15]
			P25–N33 29.0 ± 17.9 μV		
BAFME (FAME)	Variable ~100%	N20–P25 markedly increased; P25-HFOs superimposed	P25 17.8 ± 7.5 μV;	Time locked	Dubbioso [36]
			N35 30.1 ± 18.0 μV		
Lafora Disease	~75%	Both N20–P25 and P25–N33 markedly elevated	N20–P25 14 ± 10.1 μV;	No identifiable time-locked correlate with myoclonus	Canafoglia [51]
			P25–N60 33.5 ± 20.5 μV		
Sialidosis	~100%	N20–P25, mid-latency	NA	Usually present	Hsueh [52]
MERRF	~100%	Enlarged early cortical SEPs	P1–N2 13–30 μV	Not always	Thompson [53]

**Table 3:** Giant SEPs in non-epileptic disorders: clinical context.

Disorder/Etiology	Possible Interpretation	Myoclonus/EEG Correlation
Creutzfeldt–Jakob disease (CJD)	Cortical disinhibition followed by degeneration	Variable correlation with gSEP may vary with disease progression
Progressive Supranuclear Palsy (PSP)	Subcortical disinhibition	Rare or absent
CNS (central nervous system) Tumors	Focal cortical disinhibition	Rarely reported
Vascular causes (Stroke, etc)	Contralateral, possibly maladaptive or compensatory mechanism	Absent
Multiple Sclerosis (MS)	Maladaptive plasticity	Rare
Cervical myelopathy	Altered afferent conduction/cortical reorganization	Absent
Hypocalcemia (post-thyroidectomy)	Reversible cortical disinhibition	No reported
Vitamin B12 deficiency	Metabolic encephalopathy with hyperexcitability	Not specified
Hydroxychloroquine toxicity	Drug-induced cortical dysfunction	Not specified
Functional Neurological Disorders (FND)	Maladaptive cortical sensitization	Absent
Motor Neuron Disease (ALS)	Widespread cortical dysfunction	Absent
Neuropathic pain/central sensitization	Plasticity in the somatosensory cortex and afferents	Absent
Peripheral Neuropathies (e.g., anti-MAG, diabetic)	Sensory deafferentation and compensatory plasticity	Absent
Spinal cord tumors/intramedullary lesions	Cortical response to altered spinal afferents	Very Rare

## Data Availability

Not applicable.
